# Soil microbial networks’ complexity as a primary driver of multifunctionality in photovoltaic power plants in the northwest region of China

**DOI:** 10.3389/fmicb.2025.1579497

**Published:** 2025-04-22

**Authors:** Liuqing Zhao, Sumeng Xu, Jinmei Zhao, Shujuan Chen, Xiaolong Liu, Xiuyuan Zheng, Xiuhui Wang, Zhao Zhu, Fei Gao, Bingzhe Fu, Shuxia Li

**Affiliations:** ^1^Natural Resources Assets Statistics and Accounting Center of Ningxia Hui Autonomous Region, Yinchuan, Ningxia, China; ^2^College of Grassland Agriculture, Northwest A&F University, Xianyang, Shaanxi, China; ^3^Horticultural Technology Extension Station of Ningxia Hui Autonomous Region, Yinchuan, Ningxia, China; ^4^Natural Resources Information Center of Ningxia Hui Autonomous Region, Yinchuan, Ningxia, China; ^5^College of Forestry and Prataculture, Ningxia University, Yinchuan, China

**Keywords:** photovoltaic power plants (PVs), bacterial diversity, fungal diversity, microbial network complexity, soil multifunctionality

## Abstract

**Introduction:**

Exploiting photovoltaic power generation as a novel source of clean energy has become increasingly common in recent times. Nevertheless, the impact of photovoltaic power plants (PVs) on soil microbial activity and several functions is unclear.

**Methods:**

The present investigation aims to collect soil samples from photovoltaic power plants in arid and semi-arid regions with different years of construction, determine the physicochemical properties of the soil, and employ high-throughput sequencing to obtain 16S rRNA and ITS genes from the PV. This approach examines the community composition of bacteria and fungi in plant soils. This dataset is adopted to explore the role of soil physicochemical characteristics and climatic factors in the variousness and complexness of the network of soil microbial communities in PVs.

**Results:**

The findings reveal that soil physicochemical properties exhibit a gradual increase over time, with bacterial and fungal diversity showing a corresponding gradual increase and reaching a maximum over a period of 5–10 years. Furthermore, it is observed that the topological properties of the microbial network underwent significant changes driven by microbial diversity. Bacterial and fungal diversity as well as network complexity also display positive and negative correlations, respectively. A positive and significant correlation is detected between the bacterial network complexity and the soil multifunctionality, whereas a substantial negative correlation is observed between the fungal network complexity and the soil multifunctionality.

**Discussion:**

In conclusion the environment is able to directly regulate soil microbial diversity, thereby affecting network complexity and driving soil multifunctionality. Such discoveries are aimed to have crucial ecological implications for predicting environmental-soil-microbial effects on soil multifunctionality in photovoltaic zones.

## 1 Introduction

As renewable energy development accelerates globally, more photovoltaic power plants (PVs) are being constructed in desert areas to satisfy the surging demand for sustainable energy (Xia et al., 2022). Currently, the construction of PV plants is mainly concentrated in arid and semi-arid regions, because these lands are of interest for many PV applications due to their high solar potential (Thorne et al., 2015). Photovoltaic power converts solar energy into electricity with zero-carbon emissions, utilizing renewable resources to reduce reliance on fossil fuels and support global climate goals. However, while the construction of photovoltaic power stations contributes to the development of renewable energy, it also poses a series of new challenges to the soil ecological environment. Firstly, during the construction phase, site leveling and infrastructure development directly disrupt surface vegetation and biological soil crusts, leading to alterations in soil structure and degradation of ecosystem functions (Grodsky and Hernandez, 2020). Secondly, during the operational phase, the installation of PV panels alters local microclimatic conditions, creating a “rain shadow effect” that significantly reduces rainfall in the inter-panel areas and exacerbates diurnal temperature fluctuations (Barron-Gafford et al., 2016). These changes further impact soil moisture balance, vegetation growth, and carbon cycling processes. Additionally, while the shading effect of PV panels may promote the growth of certain shade-tolerant plants, it generally inhibits photosynthesis in most plants, leading to a decline in vegetation coverage and carbon sequestration capacity (Wu et al., 2010; Araki et al., 2017). These ecological impacts are particularly pronounced in arid regions, where ecosystems are inherently fragile and have limited recovery capacity.

Currently, the conclusions regarding the impact of PV power station construction on soil are not consistent. Research indicates that soil moisture content under PV panels in the Mojave Desert is significantly reduced, while studies by Li et al. (2018), Wu et al. (2022) suggest that PV panels can effectively retain soil moisture and alleviate thermal stress. Liu et al. (2019) demonstrated that surface cover, biomass, and species richness are significantly higher in the presence of PV panels compared to undisturbed environments. Liu et al. (2019) found that soil moisture content, organic matter, available potassium, and available phosphorus within PV power stations are significantly higher than in surrounding areas, although pH levels are lower. Conversely, other studies have noted that soil bulk density under PV panels increases compared to unshaded areas, while soil moisture content, available phosphorus, and available potassium content decrease (Yue et al., 2021). Therefore, a better understanding of the multifunctionality of soil in PV power stations is essential to address these environmental changes. However, the overall impact of photovoltaic panels on soil ecological environment still needs to be studied.

The sustainable development of soil multifunctionality serves as the foundation for ecosystem services, with soil microorganisms being one of the driving factors of soil multifunctionality. Clarifying the relationship between soil microorganisms and soil multifunctionality is a prerequisite for understanding the impact of photovoltaic (PV) systems on soil multifunctionality (Delgado-Baquerizo et al., 2017). Numerous studies have demonstrated that soil microorganisms play a critical role in maintaining soil multifunctionality, including organic matter decomposition, nutrient cycling, and primary production. Research indicated that even subtle changes in soil microbial communities can significantly influence soil multifunctionality (Garland et al., 2021; Fan et al., 2023). Furthermore, interactions among microorganisms—specifically, the complexity of microbial networks—also play a vital role in regulating soil functions. Wagg et al. (2019) experimentally demonstrated that a reduction in the complexity of underground microbial networks negatively affects soil multifunctionality. Additionally, studies have shown that microbial community composition is a significant predictor of soil multifunctionality (Ma et al., 2022). In recent years, discussions on the ecological impacts of PV power stations have garnered considerable attention, primarily focusing on surface solar radiation, air temperature, humidity, wind speed, and wind direction. However, there is limited research on how PV power stations influence soil microorganisms and soil multifunctionality. Therefore, exploring the effects of PV power stations on soil ecological environments and multifunctionality is of significant importance.

To this end, we herein conducted an investigation on soil PVs (microbial biodiversity-soil multifunctionality) with different construction times in Ningxia Province, Northwest China. The investigation aims to address the following critical issues: How do soil microbial diversity and soil multifunctionality of PVs differ during various construction phases? How do environmental predictions directly and indirectly affect the multifunctional relationships of soil microbial diversity? Through research efforts, this work aims to test the following hypotheses: (i) PVs modify soil microbial diversity through changes in soil properties by overshadowing; (ii) As solar construction time increases, soil microbial networks under PVs change dramatically; (iii) Regulation of microbial diversity in shaping of multifunctional soil is mainly performed due to the complexity of the network. The findings of this study will elucidate the response mechanisms through which soil microbial community diversity and structural complexity regulate soil multifunctionality within photovoltaic power plants ecosystems. This work further provides scientific basis for maintaining the stability of soil multifunctionality in photovoltaic power plant ecosystem.

## 2 Materials and methods

### 2.1 Sampling sites and methods

**FIGURE 1:**
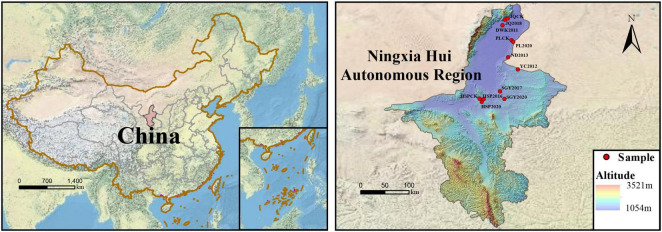
Sampling site locations, the sites spanned a longitudinal range from 106°E to 107°E and a attitudinal range from 37°N to 40°N.

The sites were collected based on the construction times of PVs and soil type from various regions of Ningxia. In total, 12 sampling sites were mainly located in five districts of Ningxia Province (38°42′04″–38°46′43″N and 105°53′50″–106°00′03″E), northwest China, and they were classified into Dawukou (DWK), Pingluo (PL), Jianquan (JQ), Yinchuan (YC), Ningdong (ND), Hongsipu (HSP), and Shigouyi (SGY) from south to north. The locations and basic climate information of the sampling sites are presented in the Supporting Information (Figure 1 and Table 1). All soil samples were haphazardly collected at each of the 12 sites using uniform sampling protocols. Each of the 12 sites included three 10 m × 10 m plots. In each plot, the samples included five soil cores (depth 0–20 cm and diameter 5 cm). Each soil core is situated directly beneath the photovoltaic panel, and the position of each sampling point relative to the photovoltaic panel is consistent. The samples were appropriately mixed, and roots were methodically eliminated, carefully sealed in bags, and swiftly moved to the laboratory on dry ice. Afterward, the soil was sieved through a 2 mm mesh to eliminate observable roots, residues, and stones. The samples were then meticulously combined to assemble a composite, and then split into three sub-samples for thorough analysis. One sub-sample was stored under 4°C conditions to ensure accurate soil enzyme activity measurements. Another sub-sample underwent natural air-drying as well as 1 mm and 0.25 mm sieving to prepare for in-depth physicochemical property analyses. The third sub-sample was frozen at –80°C to preserve its integrity for subsequent DNA analyses. The meteorological data (2013–2023) for 12 sampling locations were extracted from the National Meteorological Science Data Center. We collected average annual temperature and precipitation data for the last 10 years.

**TABLE 1:** The photovoltaics (PVs) construction time of sampling sites.

Districts	< 5 years	5–10 years	> 10 years	Undisturbed region (UR)
SGY	2020	2017	–	–
HSP	2020	2016	–	CK
ND	–	–	2013	–
YC	–	–	2012	–
JQ	–	2018	–	CK
PL	2020	–	–	CK
DWK	–	–	2011	–

### 2.2 Assessment of soil parameters and functions

Tests were conducted for soil pH, soil water content (SWC), soil organic matter (SOM), total carbon (TC), total nitrogen (TN), nitrate nitrogen (NO_3_^–^-N), ammonium nitrogen (NH_4_^+^-N), total phosphorus (TP), available phosphorus (AP), total potassium (TK), available potassium (AK), soil organic carbon (SOC), microbial biomass C (MBC), microbial biomass N (MBN), soil organic matter (SOM) as previously described (Chen et al., 2020). The potential activities of extracellular hydrolytic enzymes α-glucosidase (αG), β-1,4-glucosidase (βG), cellobiohydrolase (CBH), and N-acetyl-β-glucosaminidase (NAG) acid phosphatase (ACP), were analyzed via a fluorometric approach (Ma et al., 2020; Rui et al., 2022).

We obtained data for 12 soil variables including NO_3_^–^-N, NH_4_^+^-N, TN, TC SOC, TP, AP, MBC, MBN, βG, NAG, and ACP. These functions serve as excellent indicators of the intricate processes involved in carbon, nitrogen, and phosphorus cycling within mountain ecosystem soils. Furthermore, they play a pivotal role in capturing sequestering nutrients, promoting productivity, and enhancing soil fertility (Yang et al., 2023b). To obtain a quantitative soil multifunctional index for each sample, we first calculated the Z-score of the 12 soil properties and then averaged these transformed variables (Maestre et al., 2012; Jiao et al., 2021).

### 2.3 Soil DNA extraction and PCR amplification

The soil samples’ microbial genomic DNA was carefully extracted using the state-of-the-art E.Z.N.A soil DNA kit (Omega Bio-tek, Norcross, GA, United States) as per the manufacturer’s instructions. By doing so, the DNA quality and concentration were meticulously evaluated by adopting 1.0% agarose gel electrophoresis and a NanoDrop 2000 spectrophotometer (Thermo Scientific, United States) before being stored at –80°C for future use. Regarding the amplification process, the V5–V7 hypervariable region of the bacterial 16S rRNA gene was appropriately targeted with primer pairs 338F (5′-ACTCCTACGGGAGGCAGCAG-3′) and 806R (5′-GGACTACHVGGGTWTCTAAT-3′). Additionally, the fungal ITS2 region was amplified using the primer pair ITS1F (5′-CTTGGTCATTTAGAGGAAGTAA-3′)/ITS22R (5′- TCCTCCGCTTATTGATATGC-3′) by T100 Thermal Cycler PCR thermocycler (BIO-RAD, United States). The PCR reaction mixture, including 4 μL 5 × Fast Pfu buffer, 2 μL 2.5 mM dNTPs, 0.8 μL each primer (5 μM), 0.4 μL Fast Pfu polymerase, 10 ng of template DNA, and ddH_2_O to a final volume of 20 μL. The specific conditions of PCR amplification cycling were carefully taken into account. The process involved an initial denaturation at 95° for 3 min, followed by 27 cycles of denaturing at 95° for 30 s, annealing at 55° for 30 s, and extension at 72° for 45 s. A single extension at 72°C for 10 min concluded the process, which was then ended at 4°C. After the PCR product was attained from a 2% agarose gel and purified using the PCR Clean-Up Kit (YuHua, Shanghai, China) following the manufacturer’s instructions, it was quantified using Qubit 4.0 (Thermo Fisher Scientific, United States).

The purified DNA fragments, or amplicons, were combined in equimolar amounts to ensure equal representation of each fragment in the sequencing library. Subsequently, the library was subjected to paired-end sequencing on an Illumina PE300 platform (Illumina, San Diego, United States), which is known for its high-throughput and accuracy in DNA sequencing. The sequencing was conducted according to the standard protocols by Majorbio Bio-Pharm Technology Co. Ltd., a reputable company based in Shanghai, China, known for its expertise in genomic services. This approach ensured comprehensive and accurate sequencing of the DNA fragments, providing valuable insights into the genetic information contained within the samples.

### 2.4 Amplicon sequence examination

After separating the sequences, we meticulously filtered them for quality via fastp (ver.0.19.6) (Chen et al., 2018) and expertly combined them with FLASH (version 1.2.7) (Magoč and Salzberg, 2011). Next, we harnessed the power of the DADA2 (Callahan et al., 2016) plugin in the Qiime2 pipeline (Bolyen et al., 2019) to meticulously remove any noise from the high-quality sequences, achieving single-nucleotide resolution on the basis of the error profiles in the samples. These denoised sequences, the so-called amplicon sequence variants (ASVs), represent the data exactness pinnacle. To ensure standardized alpha and beta diversity measures, the number of sequences from each sample was appropriately reduced to 31,342, resulting in an exceptional average Good coverage of 98.85%. Furthermore, the taxonomic classification of the ASVs was executed with precision, utilizing the Naive Bayes consensus classification classifier adopted in Qiime2 and the esteemed SILVA 16S rRNA database (ver.138).

### 2.5 Statistical analysis

We performed one way ANOVA analysis to test the different construction times of PVs and microbial diversity (*p* < 0.05 was considered significant). Beta diversity was analyzed via principal coordinates analysis (PCoA) in accordance with the Bray-Curtis distances. A mantel test was adopted for evaluating the primary driving factors of the root traits which was then appropriately mixed with the Pearson correlation matrix. The correlations among the soil properties, climate factor, altitude, and soil microbial community (at ASV level) were determined by canonical redundancy analysis (RDA) using the “vegan” package. To enhance the presentation of our network analysis results, we pre-filter the low abundance and low-frequency groups (with an average relative abundance of less than 0.1 in at least two samplings). Following this, we conduct paired Spearman correlation calculations at the ASV level using the powerful “phych” package in R. The networks were suitably established by implementing the “igraph” package in R. In addition, Gephi (0.10.1) was utilized to visualize each network. We computed the topological properties of the subset network and then proceeded with calculating the network complexity of the subnetwork. Adopting the linkage density, the network complexity factor could be reasonably denoted (links per ASV) (Wagg et al., 2019; Chen et al., 2022). By application of the ordinary least squares (OLS) linear regression model, the relationships among the microbial diversity, network complexity, and soil multifunctionality could be appropriately examined. The environmental factors were analyzed by one-way ANOVA based on SPSS (27.0.1). Furthermore, the correlations among the soil properties, climate factors, bacterial and fungal diversity, network complexity, and soil multifunctionality via partial least squares path modeling (PLS-PM) were carefully scrutinized. This statistical methodology is incredibly valuable for demonstrating cause-and-effect relationships among observed and latent variables. Our path-based model’s path coefficients and coefficients of determination (R^2^) were rigorously validated using R (v. 4.3.2) with the plspm package (1,000 bootstraps). These path-based coefficients, also referred to as direct effects, furnish crucial insights into the strength and direction of the linear correlations among a wide range of variables. Indirect effects occur when we multiply the path coefficients between a predictor and a response variable and then add the product of all possible paths, excluding the direct effect. This helps us understand the complex interrelationships among variables and reveals the broader impact of our predictors (Ai et al., 2018; Fan et al., 2019).

## 3 Results

### 3.1 Role of PVs on microbial diversity and community composition

**FIGURE 2:**
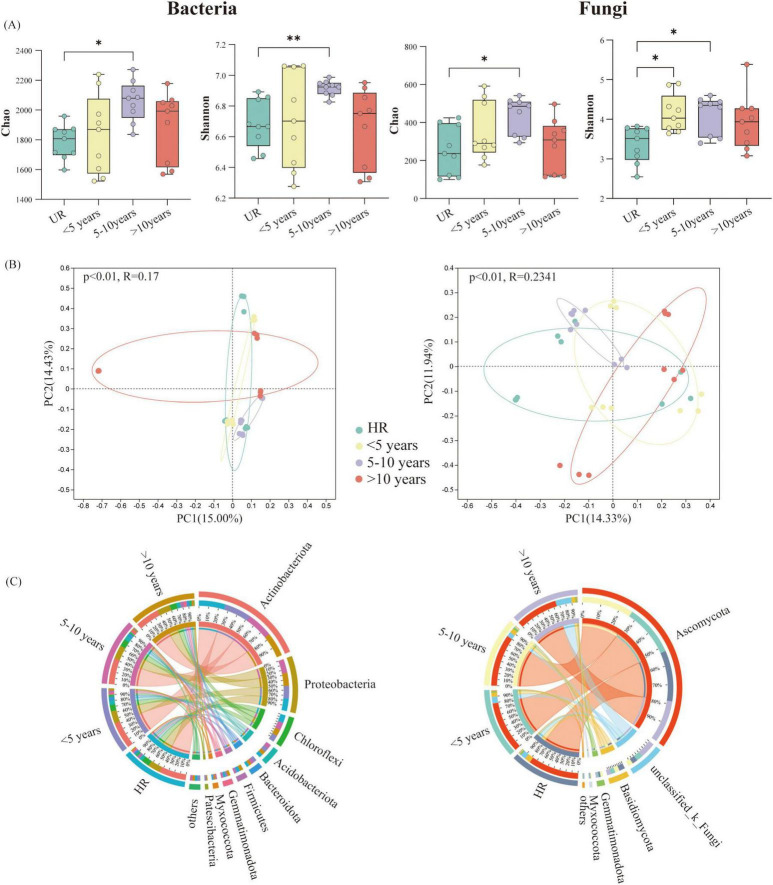
**(A)** The soil microbial alpha diversity in terms of various photovoltaics (PVs) construction times (*P < 0.05, **P < 0.01 according to the one way ANOVA besides bacterial Shannon, bacterial Shannon used Welch ANOVA). **(B)** The principal co-ordinates analysis (PCoA) on the basis of the Bray-Curtis dissimilarity matrix signifying bacterial and fungal community variations (diverse colors are indicative of dissimilar PVs construction times, and each point signifies a single replicate). **(C)** Variation in soil microbial composition among various PVs construction time (the combined enrichment ratio of the others is less than 0.01%).

A total of 2,248,021 and 2,292,919 high-quality sequences of soil bacteria and fungi were obtained. These were grouped into 39,721 bacterial ASVs and 5,468 fungal ASVs after normalizing the ASV criteria by rarefaction to an even number of sequence cut-offs. The α-diversity of PV soil bacteria and fungi were calculated in different years (Figure 2A). The α-diversity of all samples was substantially affected by different years in both bacteria and fungi. In general, α-diversity exhibited an ascending trend with the completion time of PVs, peaked for 5–10 years, and then declined. However, the variability of PVs is always higher than that of the UR. The soils collected at UR sites exhibited remarkably lower α-diversity, as estimated by the Chao and Shannon index, compared to other PVs sites. This indicates that the longer the years of PV construction, the more bacterial species were recruited within a certain timeframe (Figure 2A). The main bacterial phyla corresponding to all taken treatments were Actinobacteria, Proteobacteria, and Chloroflexi (Figure 2C). In addition, Acidobacteriota, Bacteroidota, Myxococcota, and Patescibacteria in PVs demonstrated significantly higher abundance in comparison with HR. The principal coordinate analysis (PCoA) of the Bray-Curtis distance clearly demonstrated that both the soil bacteria and fungi in PVs formed four distinct clusters in different years, as illustrated in Figure 2B. This compelling evidence indicates that the bacterial and fungal community was substantially affected by the varying conditions in various years of PV.

### 3.2 Soil physicochemical properties and enzyme activity affected by PVs

**FIGURE 3:**
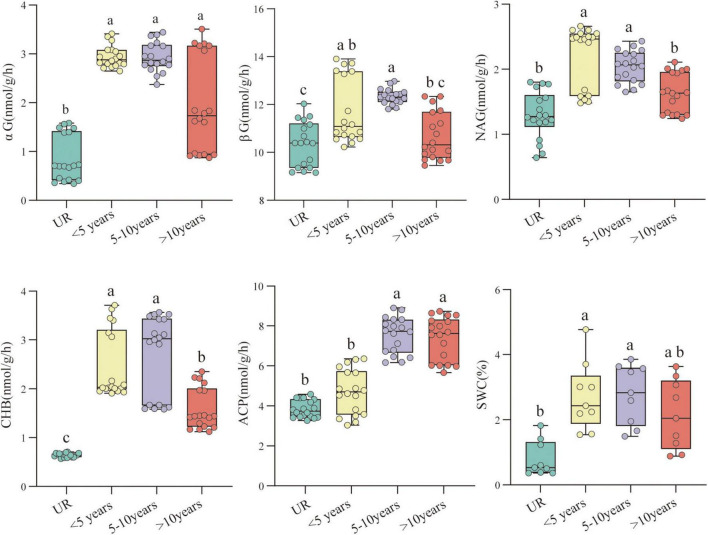
The soil enzyme activity and the soil water content as affected by various photovoltaics (PVs) construction times. Mean values with different letters are significantly different at *P* < 0.05. These are evaluated based on the Kruskal-Wallis tests).

A total of 10 out of 14 physicochemical properties of testis soil were significantly different between years of constructed PV (Supplementary Table 1). The levels of SWC, TN, NO_3_^–^-N, NH_4_^+^-N, TC, SOC, MBC, and SOM of PVs soil were substantially higher than UR. For most soil physicochemical properties, PVs of 5–10 years demonstrated a remarkable difference between less than 5 years and more than 10 years, but most soil physicochemical properties did not reach a significant level among the construction time. Five soil enzymes were substantially different between years of PV construction and UR; in addition, the soil enzyme activity exhibited an ascending trend and then a descending trend as the year of construction of PVs progressed (Figure 3).

### 3.3 Edaphic and climatic factors mediated shift in microbial community

**FIGURE 4:**
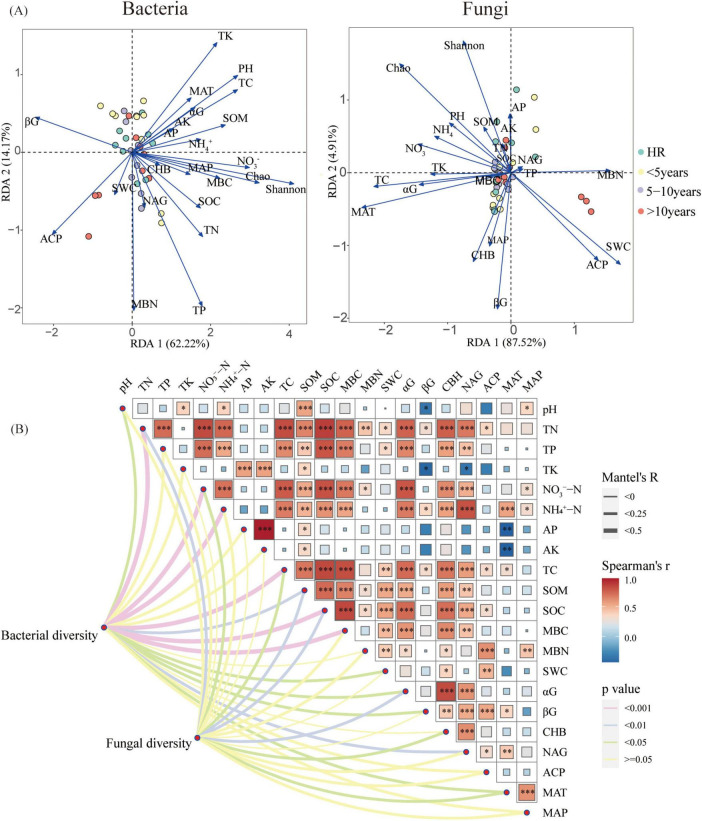
**(A)** Redundancy analysis (RDA) adopted for examining the correlations among the microbial communities of bacteria and fungi and the environmental factors; **(B)** Correlation analysis of environment factors (i.e., soil properties, climate factors, rhizosphere microbial diversity) and Mantel test of root traits and environment factors. The partial Mantel’s r statistic is represented by line width, and the color of the line indicates the statistical significance (The symbols *, **, and *** in order is indicative of the significance levels at *p* < 0.05, *p* < 0.01, and *p* < 0.001). αG: α-glucosidase, βG:β-1,4-glucosidase, CBH: cellobiohydrolase, NAG: N-acetyl-β-glucosaminidase, ACP: acid phosphatase.

Redundancy analysis (RDA) was implemented to edaphic and meteorological factors’ mediated shift in microbial assemblages with the alteration in construction time (Figure 4A). Soil pH (R^2^ = 0.32, *p* < 0.01), TP (R^2^ = 0.26, *p* < 0.05), TK (R^2^ = 0.26, *p* < 0.01), NO_3_^–^-N (R^2^ = 0.35, *p* < 0.01), TC (R^2^ = 0.30, *p* < 0.01), SOM (R^2^ = 0.22, *p* < 0.05), MBC (R^2^ = 0.20, *p* < 0.05), SWC (R^2^ = 0.21, *p* < 0.01), βG (R^2^ = 0.24, *p* < 0.05), and ACP (R^2^ = 0.20, *p* < 0.05) were significantly related to the bacterial relative abundance. Soil TC (R^2^ = 0.18, *p* < 0.05) and the mean annual temperature (MAT) (R^2^ = 0.23, *p* < 0.05) were significantly related to the fungal relative abundance.

Mantel test is mainly aimed to reveal the variation of the microbial community diversity due to the environmental factors (Figure 4B). As is seen, the bacterial diversity was correlated with pH, TN, NO_3_^–^-N, NH_4_^+^-N, TC, SOM, SOC, MBC, SWC, αG, βG, NAG, and MAT. Additionally, the results are indicative of the fact that the fungi diversity was correlated with TN, NO_3_^–^-N, TC, SOM, SOC, αG, CHB, and MAT.

### 3.4 PVs soil microorganism ecological co-occurrence assessment

**FIGURE 5:**
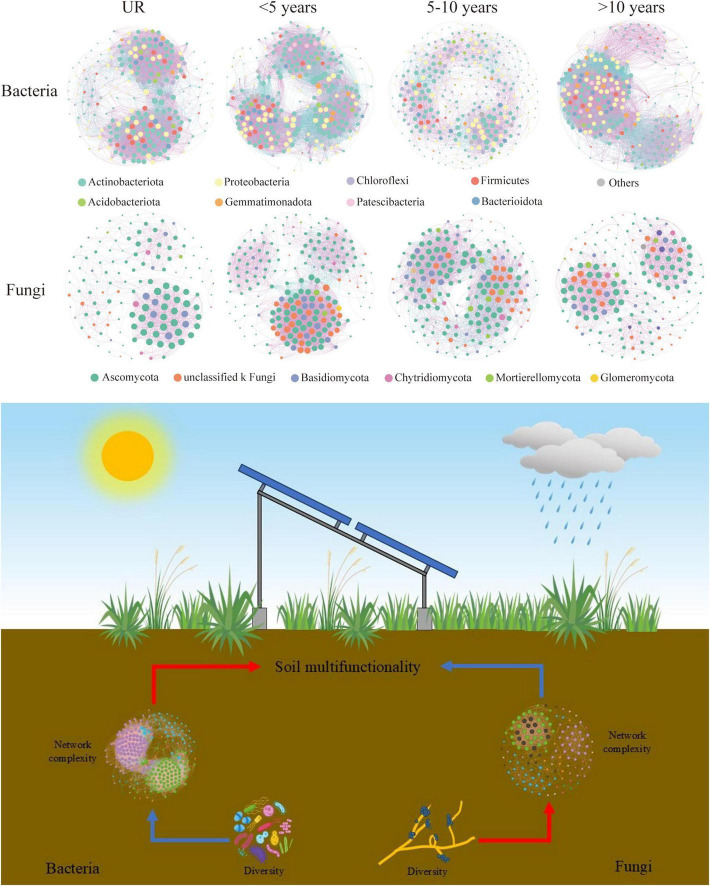
Co-occurrence networks associated with microbial communities for various photovoltaics (PVs) construction times (the dissimilar phylum in microbial networks is illustrated in dissimilar colors. The presented nodes stand for phylum, whereas the edges signify the existence of a significant correlation between nodes. The pink and blue lines in order signify the positive and negative correlations. The details of network topological attributes are provided in Supplementary Table 2).

Co-occurrence networks are suitably established to elucidate microbial co-occurrence patterns and potential interactions. For this purpose, the structural and topological indices of the corresponding network were measured (Figure 5 and Supplementary Table 2a). The obtained results indicated that the complexity of the network substantially varies in the year of construction. When comparing the UR to the bacterial communities in the PVs soil, it’s clear that the latter exhibits highly intricate and interconnected networks. The PVs soil bacterial communities with an abundance of nodes and edges, along with a superior average degree, density and network complexity, except 5–10 years. Furthermore, the nodes and edges, average degree, clustering coefficient, density, and complexity of the network were higher than others over 10 years (Supplementary Table 2). As far as fungi are concerned, fungal communities in soil PVs exhibited complex and highly connected networks compared to UR, but < 5 years included nodes, edges, degree, clustering coefficient, as well as density, and network complexity (Figure 5 and Supplementary Table 2b). As the construction times of PVs increases, the nodes, edges, degree, density of PVs gradually lessen.

### 3.5 The relationships among the microbial diversity, network topology, and soil multifunctionality

**FIGURE 6:**
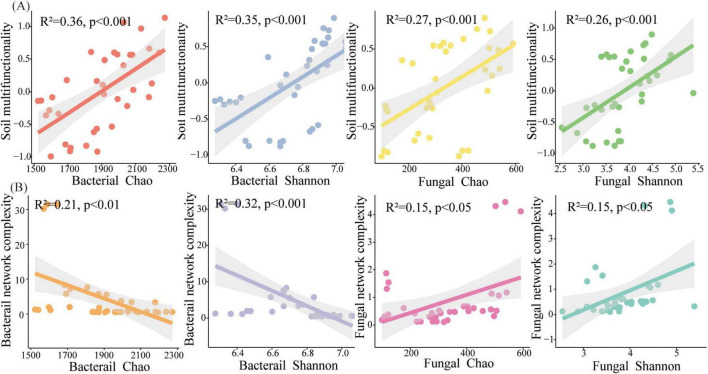
**(A)** Soil multifunctionality as a function of the bacterial and the fungal diversity; **(B)** Bacterial network complexity as a function of the bacterial and the fungal diversity (gray shading signifies 95% confidence intervals).

**FIGURE 7:**
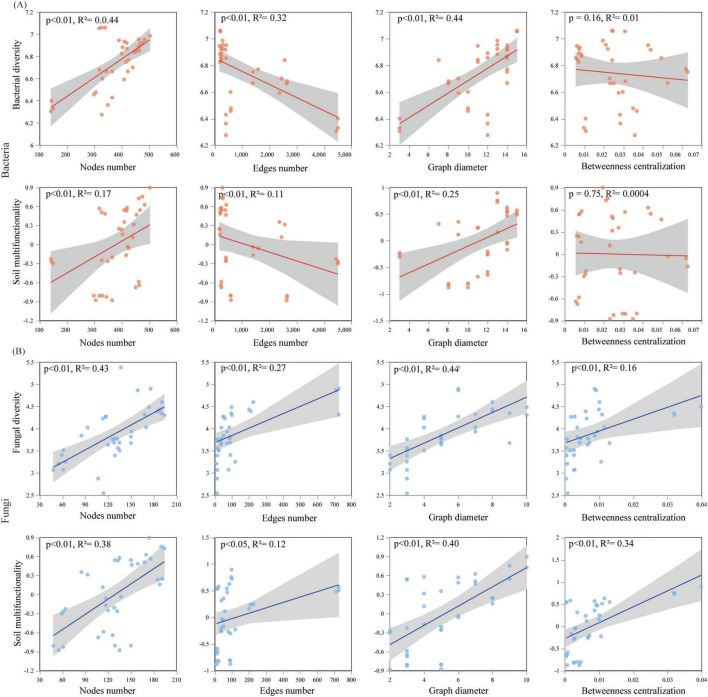
Relationships among: **(A)** bacterial, **(B)** fungal for the network topological properties as well as diversity and soil multifunctionality (gray shading indicates 95% confidence intervals).

The relationships among the microbial diversity, network completeness, and soil multifunctionality were methodically explored (Figure 6). The obtained results are indicative of the fact that a significant positive relationship between the bacterial Chao index and the soil multifunctionality is observable (R^2^ = 0.37, *p* < 0.05), and the bacterial Shannon index was related to the soil multifunctionality (R^2^ = 0.35, *p* < 0.001) (Figure 6A). Regarding fungi, Chao (R^2^ = 0.27, *p* < 0.001) and Shannon (R^2^ = 0.26, *p* < 0.001) were significantly positive related to the soil multifunctionality (Figure 6A). The variations of the microbial diversity (Shannon) and the soil multifunctionality in terms of the topological indices have been also methodically examined (Figures 6B, 7). The bacterial diversity and multifunctionality were significantly positively related to the number and diameter of nodes and significantly negatively related to the number of edges and network complexity (Figures 6B, 7A). The fungal diversity and soil multifunctionality were significantly positively related to the number of nodes, number of edges, diameter, betweenness centrality, and network complexity (Figures 6B, 7A, B).

### 3.6 Potential drivers of PVs soil multifunctionality

**FIGURE 8:**
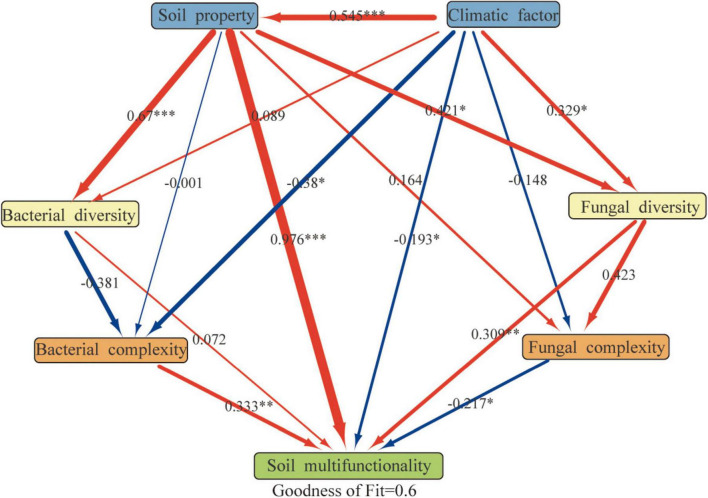
Directed graph pertinent to the partial least squares path model (PLS-PM) [individual boxes characterize detected variables (i.e., measured values) or latent variables (i.e., constructs). The path coefficients are essentially evaluated after 1,000 bootstraps and almost presented within the arrow’s width. In addition, the red arrow represents the positive effect, while the blue arrow denotes the negative effects; The symbols *, **, and *** are similar to those introduced in Figure 2, and the model’s evaluation is methodically performed via the Goodness of Fit (GoF) statistic].

Partial least squares path model SEM analysis identified integrated pathways affecting the multifunctionality of soil PVs in the northwest regions (Figure 8) (GOF = 0.6). Soil properties significantly influenced bacterial diversity (0.67, < 0.001), soil multifunctionality (0.976,< 0.001), and fungal diversity (0.421, < 0.05). The climatic factor also significantly affected soli multifunctionality (–0.193, < 0.05) and fungal diversity (0.329, < 0.05). The soil multifunctionality was significantly affected by bacterial complexity (0.333, < 0.01), fungal complexity (–0.217, < 0.05), and fungal diversity (0.309, < 0.01).

## 4 Discussion

### 4.1 The soil properties of PVs in different years have changed significantly

This study found that the soil properties changed significantly among the construction timesof PVs (Supplementary Table 1). Most of the soil properties enhance with the increase of construction time except for pH, TK, TP, TK AP, and AK. With increasing the construction time, the SWC under the PVs gradually increased. This is due to the lower evaporation potential of PVs, which promotes soil water retention and improves the effective soil water content (Liu et al., 2019). In addition, PV panels effectively reduce soil temperature and promote the growth of various species, relieving water stress that limits plant growth in arid and semi-arid regions (Niu et al., 2008). Increasing soil moisture not only directly increases plant growth, but also increases above-ground biomass by increasing community diversity (Philippot et al., 2024).

Plants and soil are inextricably linked, and soil not only holds plants in place but also provides nutrients for plant growth. The accumulation of litter under PVs has led to a substantial growth in the TN and TC. This can be essentially attributed to the presence of organic nitrogen and organic matter within the litter itself. Litter is capable of adding a large amount of organic nitrogen and organic carbon to the soil during decomposition, which is broken down by microorganisms and enzymes, increasing soil nitrogen and carbon pools, suggesting that PV control contributes to carbon sequestration. Accordingly, with the extension of the construction period of the PVs plant, significant differences in soil properties appeared, especially in the concentrations of total nitrogen (TN), nitrate nitrogen (NO_3_^–^-N), total carbon (TC), soil organic carbon (SOC), and soil organic matter (SOM). These observations are generally consistent with the results reported by Lambert et al. (2021) (Supplementary Table 1). Construction of a PV may be in its initial phase. During this period, land leveling and removal of vegetation could lead to soil damage. As the number of years of construction increases, the soil moisture content may undergo significant changes, leading to changes in the biomass of above-ground plants and thus in the nature of the soil (Lambert et al., 2021). When a certain number of years of PV construction have passed, the nutrients accumulated in the surface layer are leached into the deeper soil layers. In the absence of external recharge, this yields a reduction of the physicochemical characteristics of the soil. In addition, fencing and long-term shading also contribute to the lessening of plant diversity of the PV plant, which in turn leads to the reduction of the physicochemical characteristics of the soil (Chen et al., 2023).

### 4.2 PVs microbial composition and diversity displaying significant year differences

The diversity of the soil microbial community is crucial for preserving and assessing soil quality. High diversity enhances the resilience and adaptability of microbial processes, whereas low biodiversity impacts organic matter decomposition and nutrient cycling. The findings of the present investigation revealed a fairly remarkable disparity in the microbial diversity between the PVs and the undisturbed region (Figure 2A), which was essentially attributed to the enhancement of soil microbial community diversity due to increased inputs of soil organic matter, including litter, after PV establishment. However, microbial diversity did not increase with the number of years of construction and began to lessen after reaching a certain number of years. The shading of photovoltaic (PV) panels has been observed to increase soil moisture, which in turn enhances microbial diversity within a certain range (< 5, 5–10 years). Some microorganisms are highly adapted to grow in dry, aerobic soil conditions, and persistent increases in soil moisture may potentially inhibit the beneficial effects of soil microorganisms (Yue et al., 2021). On the other hand, the shading of photovoltaic (PV) panels resulted in a reduction in soil temperature, which may inhibit the activity and growth of microorganisms in later stages (more than 10 years) (Chen et al., 2023).

The diversity of soil microbes differed between PVs and UR. The Acidobacteriota family increased with the number of years of construction and then gradually decreased (Figure 2C). This represents a significant member of the soil microbial community and plays a pivotal role in elemental cycling and ecosystem construction. Additionally, it is responsible for the degradation of plant residues (Kanokratana et al., 2011). Consequently, in the initial phase of PVs (< 10 years), the increase in plant litter led to a growth in the abundance of the *Acidobacterium* phylum in the early stages. However, in the later stages, due to the lack of nutrients supplied by the plant, the abundance of the *Acidobacterium* phylum gradually decreased. The achieved results represented that *Bacteroidota* could exhibit a pivotal role in regulating the soil nitrogen cycle since several genera/species possess nosZ genes, which encode nitrous oxide reductase. This enzyme could assist reduce the potent greenhouse gas nitrous oxide (N2O) and improve the soil capacity for N2O pooling (Saghaï et al., 2022; Pan et al., 2023). Furthermore, the dominant phylum *Actinomycetes* was found to be significantly more predominant in PV soils than in UR soils (Figure 2C). This phylum demonstrated notable capabilities for phosphorus solubilization, potassium solubilization, nitrogen fixation, and mineralization (Huss-Danell, 1997; Shutsrirung et al., 2013). The latter process plays a pivotal role in mediating the participation of substances in biogeochemical cycling. It has been extensively documented to be beneficial for promoting plant growth and maintaining soil structure and strength (De Mandal et al., 2017; Guarino and Sciarrillo, 2017; Bao et al., 2021). *Proteobacteria* represents another main bacterial phylum in PV soils such that our findings reveal that *Proteobacteria* is more prevalent than UR in PV plant soils. It is typically regarded as a nutrient-rich bacterium with a high growth rate in carbon-rich environments (Eilers et al., 2010). In photovoltaic grassland, *Ascomycota* is the dominant taxon of soil fungi. Prior studies have shown that *Ascomycota* is commonly found in environments with high soil lignin content and is a crucial decomposer of complex compounds. This fungus is essential in the decomposition of plant residues as well as the degradation of straw residues (Bastida et al., 2016). In conclusion, the construction of PVs could significantly boost biodiversity, but this outcome depends on how long the construction process lasts.

### 4.3 Role of the PVs in altering the complexity of soil microbial networks

Microorganisms develop special interactions, such as symbiotic, competing, and predating, within specific ecological niches (Barberán et al., 2012). Soil microbial complexity of PVs varied with construction time. For instance, bacterial diversity was highest after 5–10 years, whereas the network complexity reached its lowest level at that time (Supplementary Table 2). This compelling finding highlights that shifts in soil microbial diversity may not necessarily align with alterations in microbial networks (Yang et al., 2023a), which contradicts the findings of certain studies. The uncertain variations of the diversity in terms of the network complexity clearly emphasize the significance of examining relationships within the microbiome, where the impact of individual species on network complexity does not exist independently, but is the result of positive and negative, direct and indirect linkages between various species (Wagg et al., 2019). Our results also showed the alterations in the topological properties of the networks that occur during the different years of construction of the PV installation. For example, topological attributes such as points, modularity, and network diameter of the soil bacterial co-occurrence network, as well as nodes, average degree, and density of the fungal network, show an initial increase and then a decrease with PV construction years (Figure 5 and Supplementary Table 2). This may indicate that as soil moisture and nutrients increase, bacteria and fungi dominate a pioneer taxonomic group in the early stages of PV construction. Further investigations have proven that elevated soil moisture in PV plants results in increased vegetation and greater nutrient release from apoplasts and root systems. Consequently, this fosters heightened soil microbial activity and a more intricate microbial community, thereby enhancing the plants’ ability to adapt to environmental fluctuations (Hu et al., 2022).

### 4.4 The critical role of bacteria and fungi in driving soil multifunctionality

The associations between microbial communities configure the basis of efficient transfer of energy, matter, and information within ecosystems (Faust et al., 2012). The realization of ecosystem functions (i.e., plant production, nutrient cycling, organic matter decomposition) is not the consequence of the performance of a single microbial taxon. Instead, it is driven by the synergistic action of many microbial species (Bardgett and Caruso, 2020). Recently, a proliferation of research has been conducted on examining the microbial growth traits in terms of the soil ecosystem performance. Our investigation revealed that microbial network complexity, rather than microbial diversity, was a more significant predictor of soil multifunctionality. The findings indicated that soil physicochemical characteristics, climatic factors, bacterial and fungal network complexities, and fungal diversity possess a noticeable impact on soil multifunctionality (Figure 8). There exists a reasonably good agreement between this finding’s results and the predicted results by other investigators (Delgado-Baquerizo et al., 2016). Previous research suggested that bacterial diversity may serve as a predictor of soil multifunctionality (Ying, 2023). Our findings also indicated a positive correlation between the bacterial diversity and the soil multifunctionality but this correlation did not reach a statistically significant level. Bacterial diversity exerts an indirect influence on soil multifunctionality by network complexity, whereas fungal diversity and network complexity exhibit a direct influence on soil multifunctionality. The differing influences of bacteria and fungi on soil multifunctionality may be attributed to differences in their physiology. Compared to fungal communities, soil bacterial communities essentially circulate easily degradable substrates and have easy access to nutrients such as carbon sources to facilitate their community development, they may be less resilient in the face of adversity (Li et al., 2019; Gao et al., 2022). In contrast, fungal communities are commonly more resilient. Actually, higher fungal diversity incorporates into the increase of the multifunctionality of soil in arid environments by facilitating organic matter decomposition and improving nutrient availability and soil resource allocation (Hu et al., 2021). To sum up, the construction of PVs plants could lead to the enhancement of the soil multifunctionality by growing the complexity of the microbial network.

## 5 Conclusion

The current investigation demonstrates that the construction of PVs plants exhibits a profound effect on the soil microorganisms. Furthermore, the construction of PV plants has been revealed to alter the physicochemical characteristics of the soil, which sequentially influences the soil microbial community structure. The results were indicative of the fact that the construction of PVs plant is capable of altering the topological characteristics and network complexity of the microbial network. Soil multifunctionality is strongly related to these alterations in soil microbial community characteristics, especially network complexity. Overall, the obtained results offer new insights into the prediction of soil multifunctionality in PVs.

## Data Availability

16S rRNA and ITS sequence data were deposited to the Sequence Read Archive of the NCBI database under accession no. PRJNA1244072.
