# Supplementary material for: Soil microbial networks’ complexity as a primary driver of multifunctionality in photovoltaic power plants in the northwest region of China
Source: Front Microbiol. 2025 Apr 22;16:1579497. doi: 10.3389/fmicb.2025.1579497 (PMC12052761; doi:10.3389/fmicb.2025.1579497)
Supplement: Supplementary file 1 [file Table_1.docx]

Supplementary Material

| Groups | SWC(%) | PH | TN  (g/kg) | TP  (g/kg) | TK  (g/kg) | NO_3_^-^-N  (mg/kg) | NH_4_^+^-N  (mg/kg) | AP  (mg/kg) | AK  (mg/kg) | TC  (g/kg) | SOC  (g/kg) | MBC  (mg/kg) | MBN  (mg/kg) | SOM  (g/kg) |
| --- | --- | --- | --- | --- | --- | --- | --- | --- | --- | --- | --- | --- | --- | --- |
| UR | 0.8±  0.18b | 8.51±  0.13a | 0.17±  0.01c | 0.24±  0.01b | 17.19±  0.09a | 5.51±  0.24b | 1.3±  0.09c | 1.42±  0.09a | 128±  8.49a | 10.63±  0.63c | 1.79±  0.04c | 28.66±  3.52c | 1.85±  0.21c | 2.07±  0.15b |
| <5years | 2.15±  0.35a | 8.82±  0.06a | 0.35±  0.03ab | 0.31±  0.02ab | 17.53±  0.51a | 8.17±  0.4a | 2.81±  0.29a | 1.64±  0.23a | 147.44±  20.48a | 15.24±  0.3a | 5.52±  0.57ab | 48.99±  3.51a | 2.18±  0.13c | 3.47±  0.3a |
| 5-10years | 2.78±  0.3a | 8.49±  0.11a | 0.39±  0.02a | 0.37±  0.04a | 17.04±  0.17a | 8.47±  0.62a | 2.06±  0.08b | 1.36±  0.05a | 122.22±  4.13a | 16.93±  0.17a | 6.02±  0.34a | 55.43±  0.93a | 3.07±  0.09b | 3.54±  0.25a |
| >10years | 2.72±  0.35a | 8.54±  0.09a | 0.3±  0.02b | 0.28±  0.02ab | 17.01±  0.13a | 7.49±  0.75a | 1.48±  0.23bc | 1.61±  0.07a | 145.11±  6.65a | 12.97±  0.99b | 4.45±  0.26b | 37.66±  1.75b | 4.04±  0.18a | 2.82±  0.15a |

**Supplementary table 1** Soil properties of PVs at different construction times

Note: Mean value and standard error of n = 5 biological replicates are shown. Different letters in the same column indicate significant differences (P < 0.05) among treatments. TN:soil total nitrogen, TK:soil total nitrogen, TP:soil total phosphorus, NO3--N:nitrate nitroge, NH4+-N:ammonium nitrogen, AP:soil available phosphorus, AK:soil available potassium, SOM:soil organic matter, MBC:soil microbial biomass carbon, MBN:soil microbial biomass nitrogen

| Groups | Number of nodes | Number of edges | Average degree | Modularity | Clusting coefficient | Density | Diameter | Positive ratio | network complexity |
| --- | --- | --- | --- | --- | --- | --- | --- | --- | --- |
| <5years | 279 | 8689 | 62.964 | 0.565 | 0.661 | 0.229 | 6 | 71.76 | 31.14 |
| 5-10years | 307 | 3387 | 22.07 | 0.68 | 0.52 | 0.07 | 8 | 85.95 | 11.03 |
| >10years | 245 | 9610 | 78.449 | 0.335 | 0.732 | 0.322 | 7 | 62.79 | 39.22 |
| UR | 243 | 6569 | 45.07 | 0.48 | 0.68 | 0.22 | 8 | 79.56 | 27.03 |

**Supplementary table 2a** Soil bacterial network topology indices for PVs with different construction times

**Supplementary table 2b** Soil fungal network topology indices for PVs with different construction times

| Groups | Number of nodes | Number of edges | Average degree | Modularity | Clusting coefficient | Density | Diameter | Positive ratio | network complexity |
| --- | --- | --- | --- | --- | --- | --- | --- | --- | --- |
| <5years | 199 | 3788 | 38.07 | 0.526 | 0.669 | 0.192 | 7 | 90.95 | 19.04 |
| 5-10years | 182 | 3004 | 33.01 | 0.52 | 0.59 | 0.18 | 8 | 67.24 | 16.51 |
| >10years | 172 | 1659 | 19.291 | 0.582 | 0.596 | 0.113 | 11 | 98.13 | 9.65 |
| UR | 136 | 1004 | 14.765 | 0.558 | 0.57 | 0.109 | 7 | 96.51 | 7.38 |
